# Supplementary material for: Ibrutinib Inhibits BTK Signaling in Tumor-Infiltrated B Cells and Amplifies Antitumor Immunity by PD-1 Checkpoint Blockade for Metastatic Prostate Cancer
Source: Cancers (Basel). 2023 Apr 18;15(8):2356. doi: 10.3390/cancers15082356 (PMC10136622; doi:10.3390/cancers15082356)
Supplement: Supplementary file 1 [file cancers-15-02356-s001.zip › supplementary Figures and table.pdf]

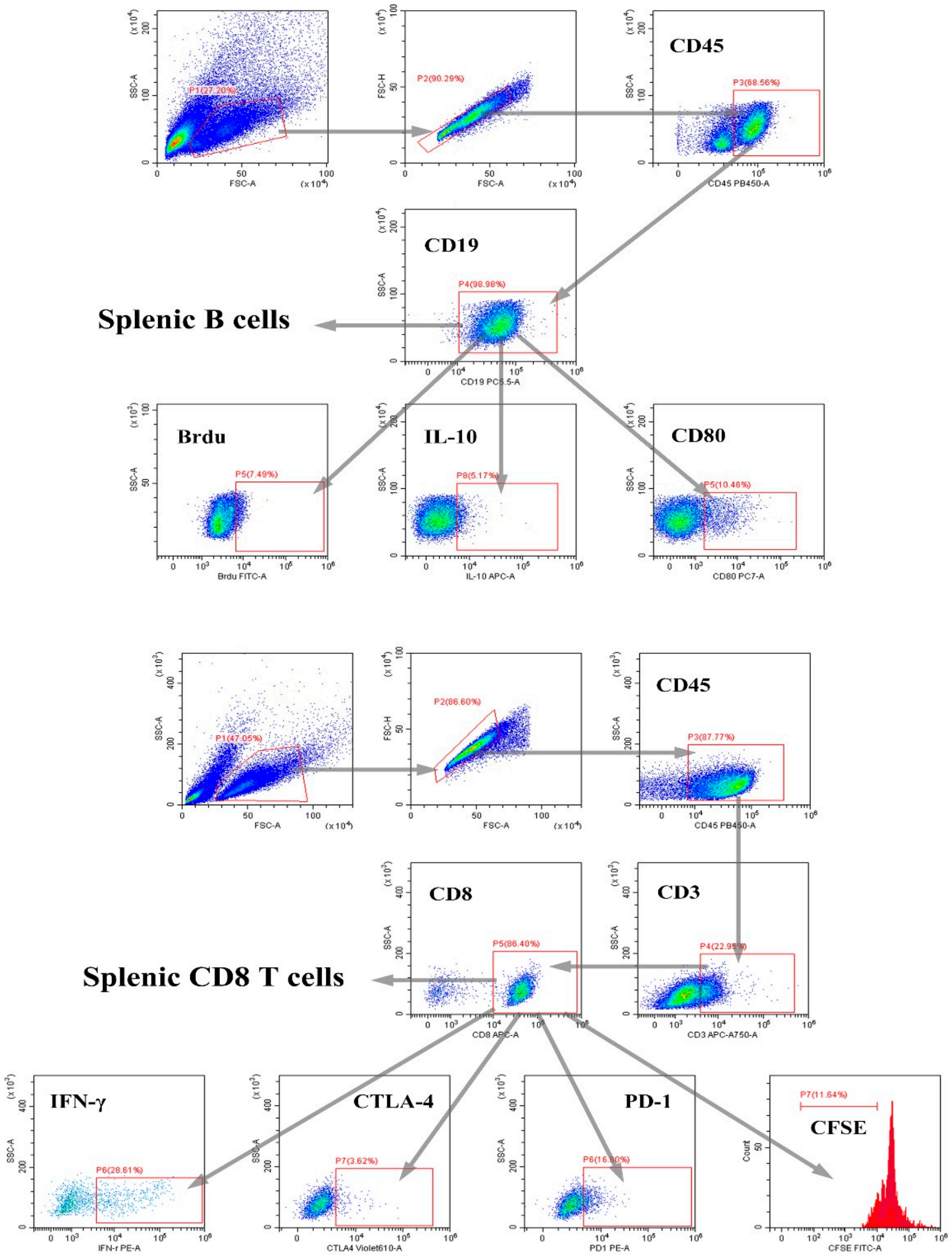

Figure S1. Gating strategy for in vitro study.

Tumor-infiltrated B cells and CD8 T cells

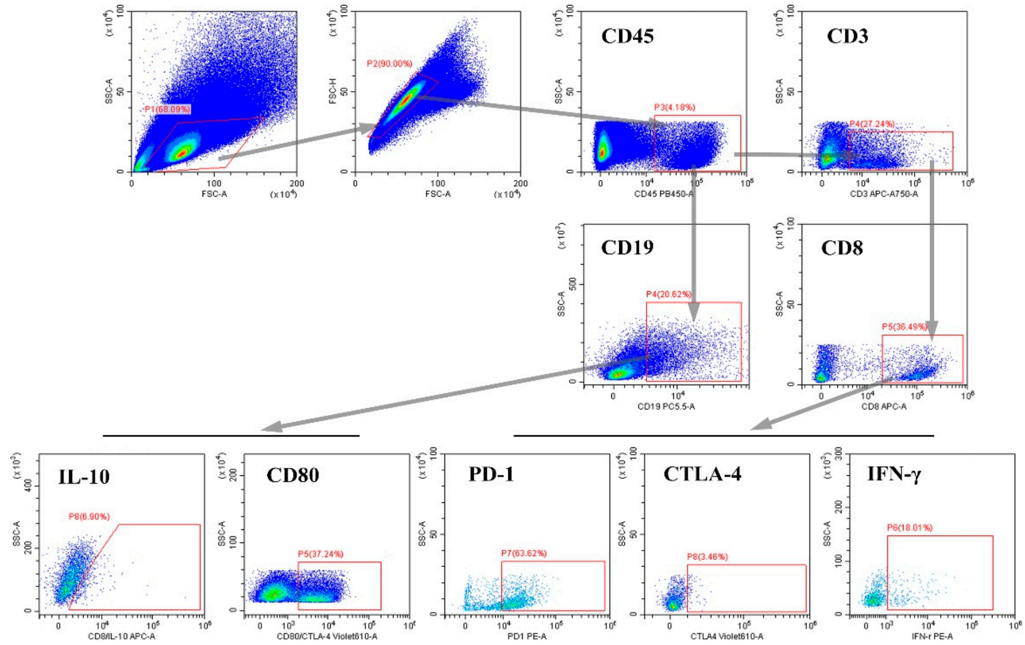

Figure S2. Gating strategy for in vivo study.

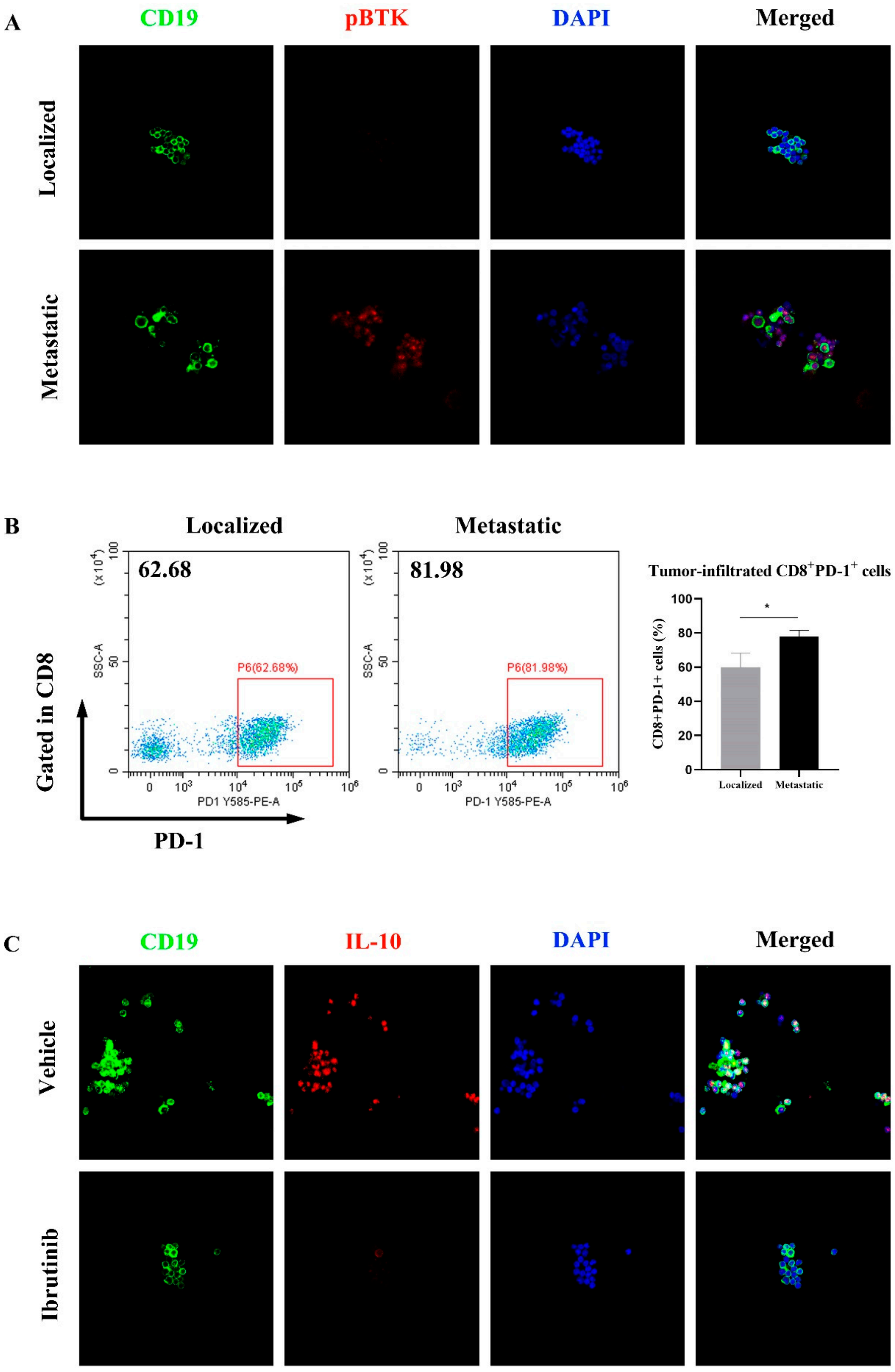

Figure S3. Validation of BTK phosphorylation and immunosuppressive molecules in tumor-infiltrating lymphocytes isolated from patients' prostate cancer tissues. (A) Representative

immunofluorescence images showing different BTK phosphorylation in tumor-infiltrating lymphocytes isolated from localized and metastatic prostate cancers (Original magnification: 400×). (B) Flow cytometry plots showing tumor-infiltrating CD8^{+}PD-1^{+} T cells were significantly higher in metastatic prostate cancers than that of in localized ones (n=3). (C) IL-10 productions in B cells purified from tumor-infiltrating lymphocytes in metastatic cancers were decreased after ibrutinib (1 μM) treatment for 24 hours (Original magnification: 400×). A p value less than 0.05 was considered statistically significant. *, $p < 0.05$.

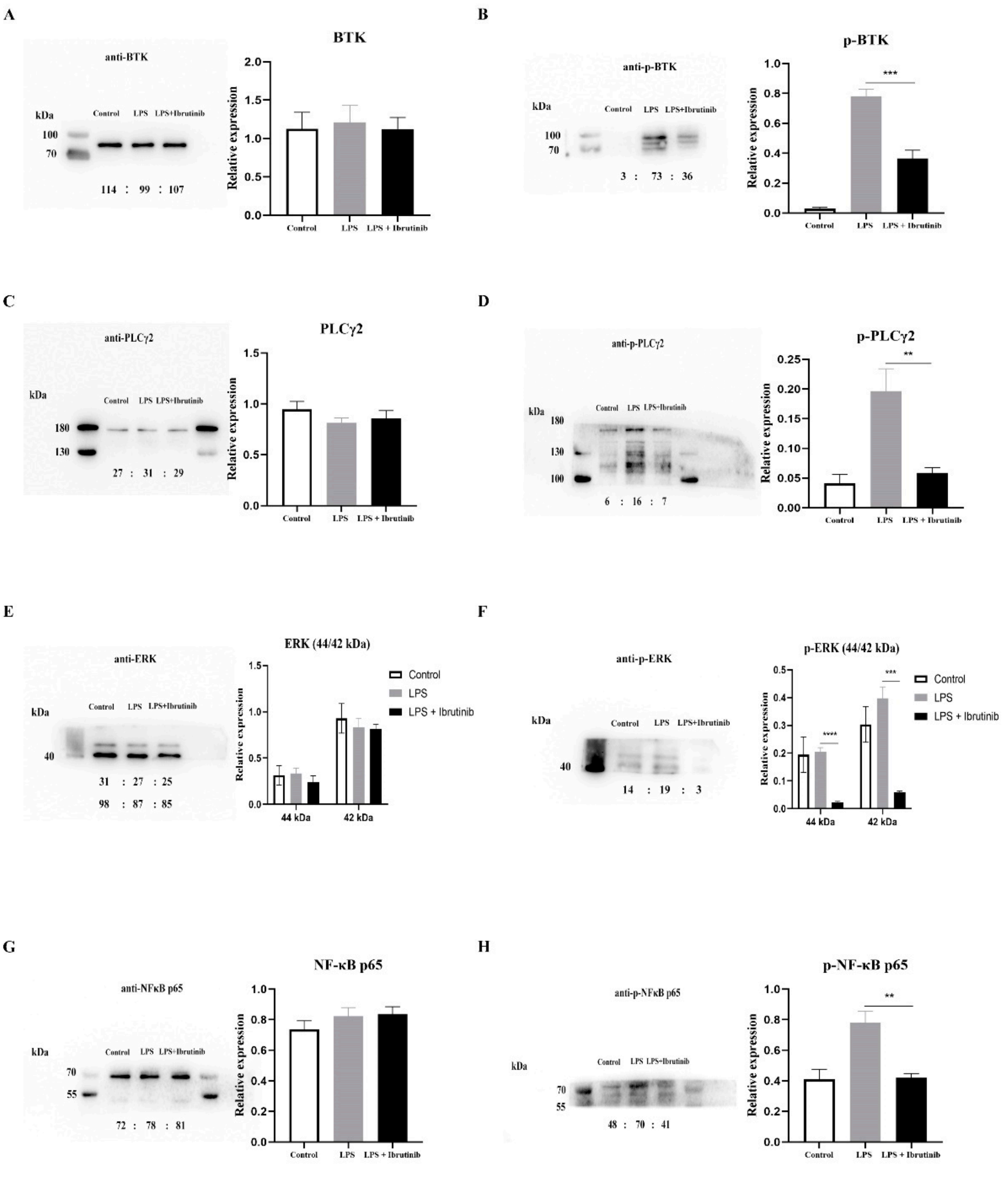

Figure S4. Western blots with molecular weight markers and intensity ratio (relative to GAPDH) of each band. A p value less than 0.05 was considered statistically significant. **, $p < 0.01$; ***, $p < 0.001$.

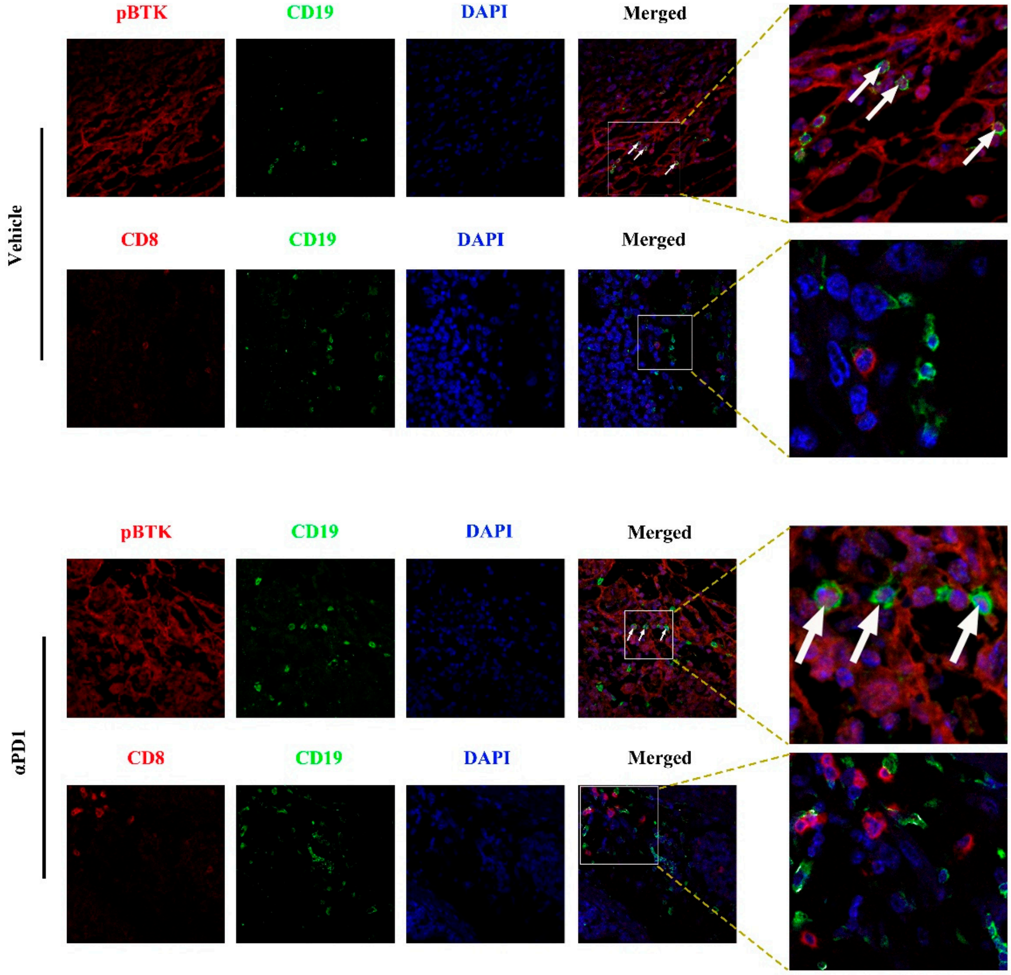

Figure S5. Representative images of BTK phosphorylation in tumor-infiltrating B cells and $CD8^{+}$ T cells infiltration in orthotopic prostate tumor of mice in αPD-1 monotherapy and vehicle groups (original magnification: 400×).

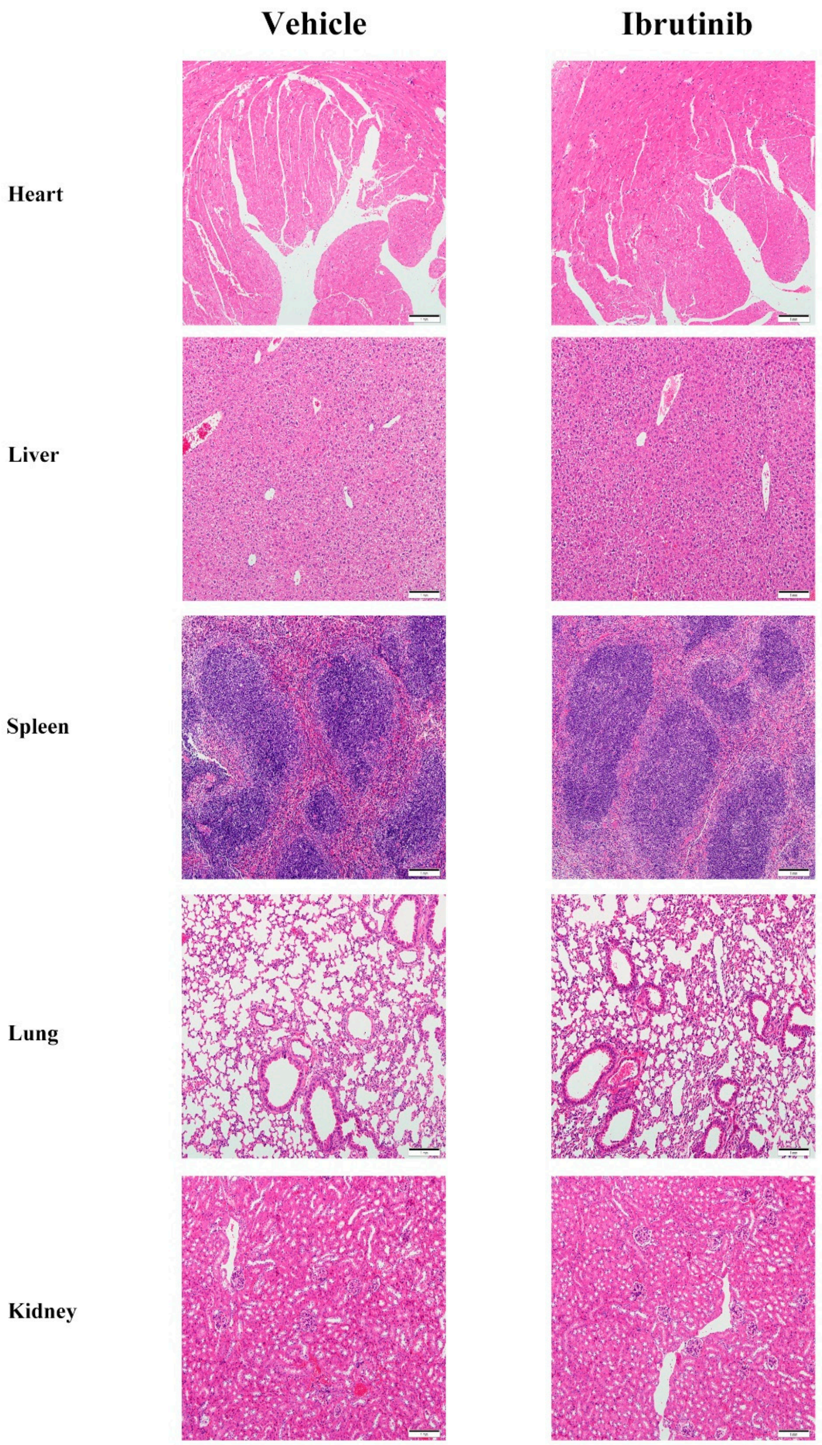

Figure S6. Pathological evaluation of mouse vital organ damage. Representative HE staining images show no pathological change in mouse hearts, livers, spleens, lungs, and kidneys after continuous ibrutinib administration (original magnification: 100×).

Table S1. Average expression of each protein relative to GAPDH in B cells detected by Western Blot.

Proteins	Intensity		
	Control	LPS	LPS + Ibrutinib
BTK	1.124	1.212	1.123
p-BTK	0.030	0.780	0.366
PLCγ-2	0.946	0.815	0.855
p-PLCγ-2	0.041	0.197	0.059
ERK (44/42 kDa)	(0.314/0.930)	(0.334/0.833)	(0.242/0.817)
p-ERK (44/42 kDa)	(0.194/0.304)	(0.205/0.397)	(0.023/0.059)
NF-κB (p65)	0.738	0.822	0.838
p-NF-κB (p65)	0.412	0.780	0.421
