# Supplementary figures and images for: Ibrutinib Inhibits BTK Signaling in Tumor-Infiltrated B Cells and Amplifies Antitumor Immunity by PD-1 Checkpoint Blockade for Metastatic Prostate Cancer
Source: Cancers (Basel). 2023 Apr 18;15(8):2356. doi: 10.3390/cancers15082356 (PMC10136622; doi:10.3390/cancers15082356)

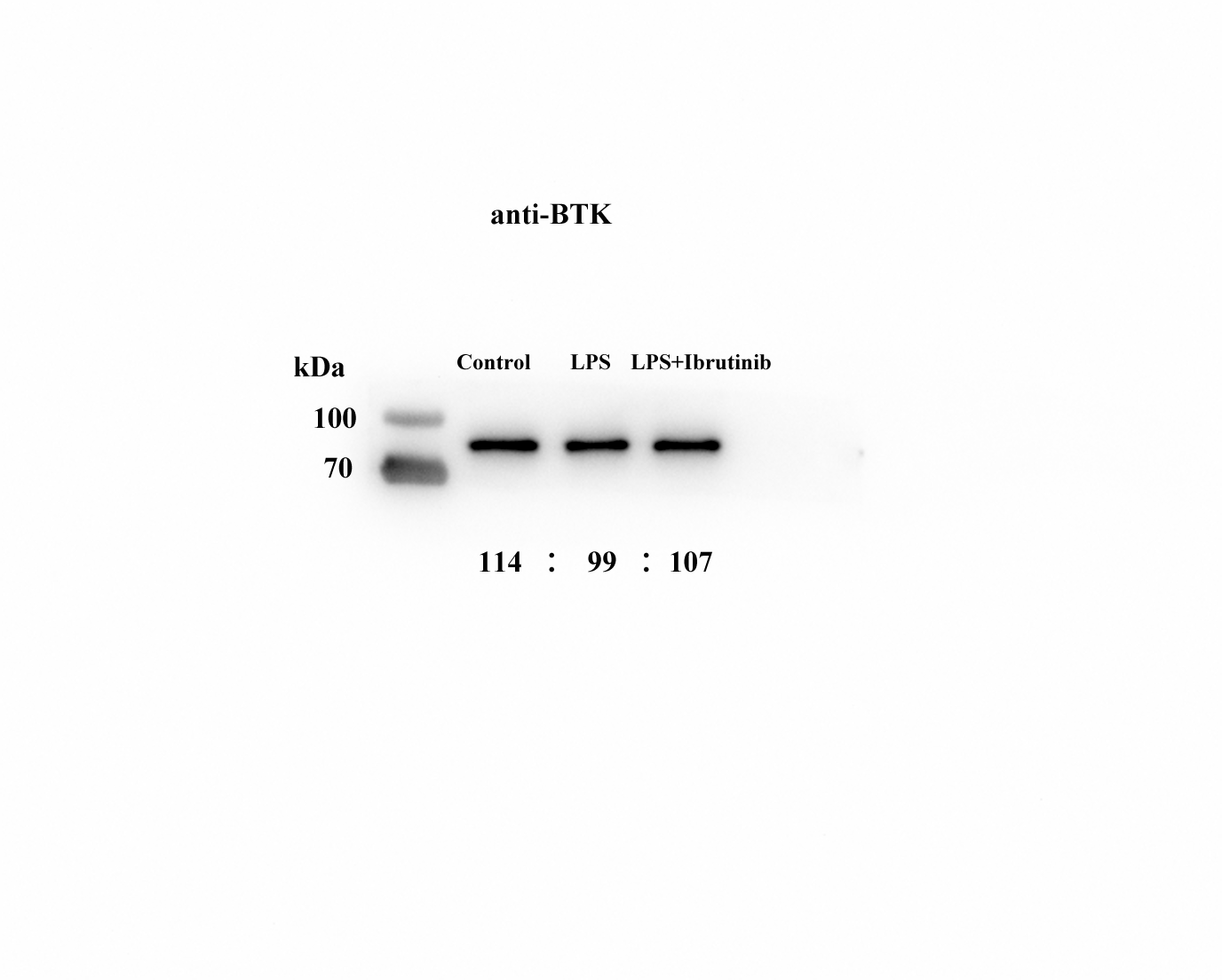

Supplement: Supplementary file 1 [file cancers-15-02356-s001.zip › file S1-Original Images for Blots/WB/BTK/BTK-vitro-1.tif]

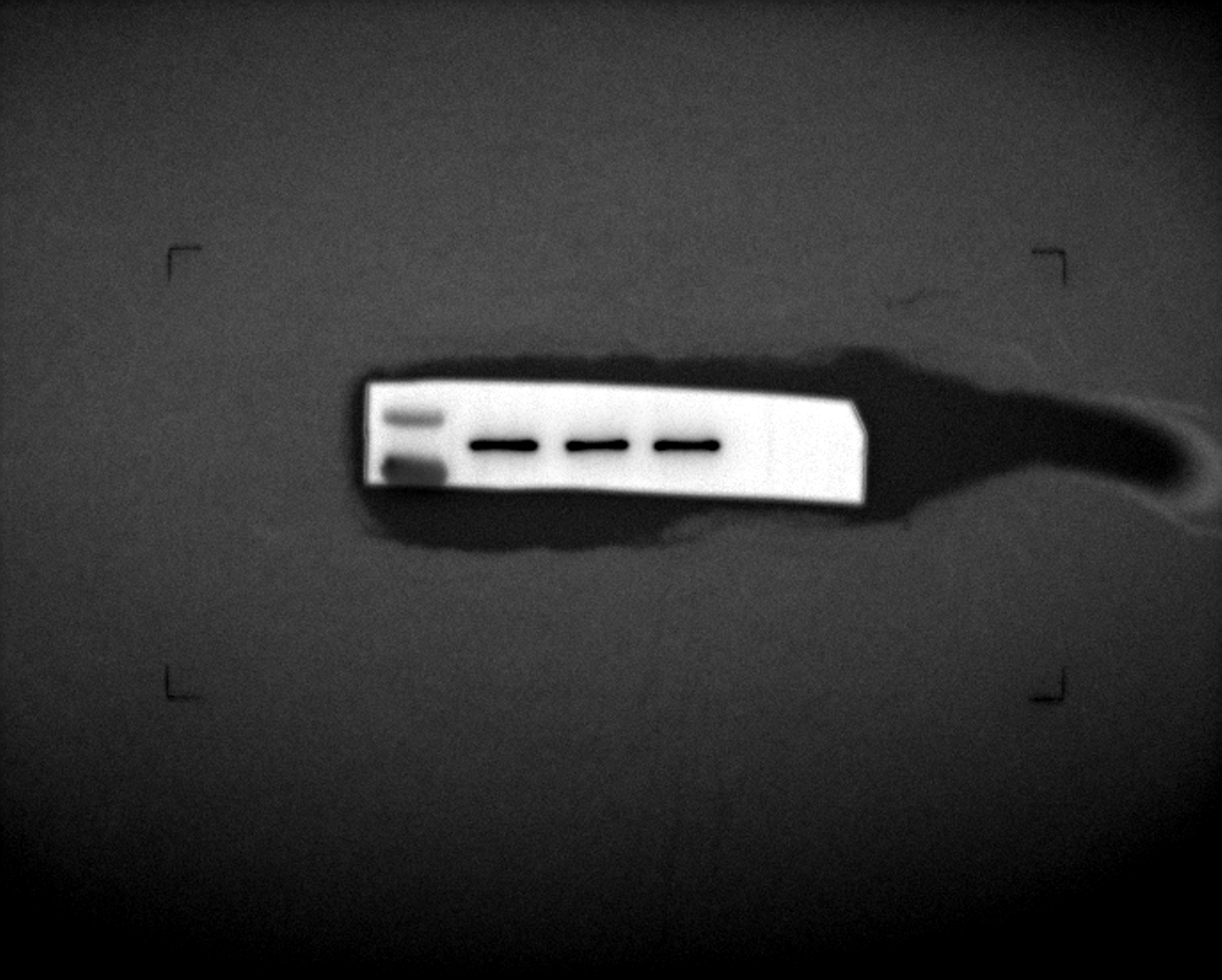

Supplement: Supplementary file 1 [file cancers-15-02356-s001.zip › file S1-Original Images for Blots/WB/BTK/BTK-vitro-2.tif]

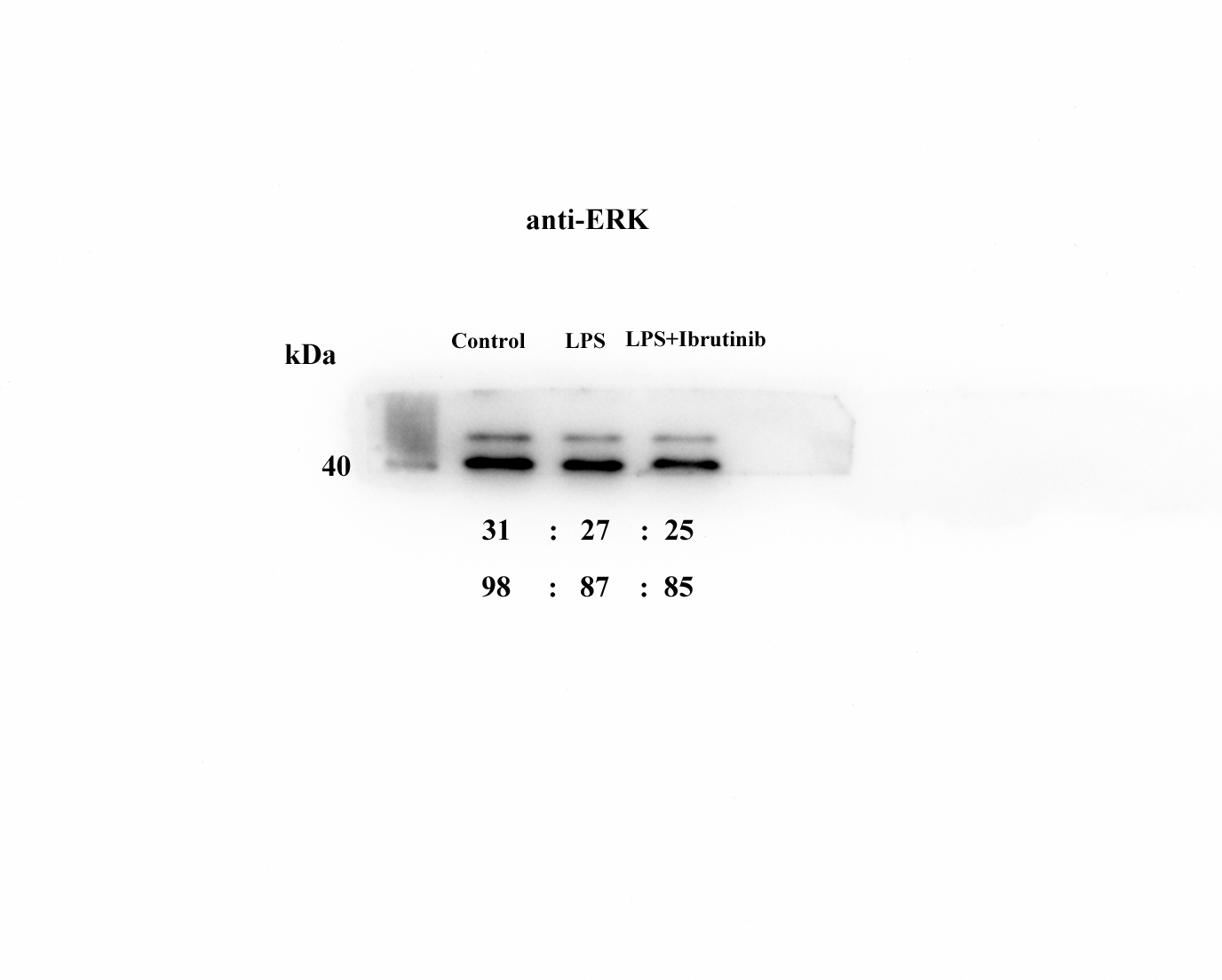

Supplement: Supplementary file 1 [file cancers-15-02356-s001.zip › file S1-Original Images for Blots/WB/ERK/ERK-vitro-1.tif]

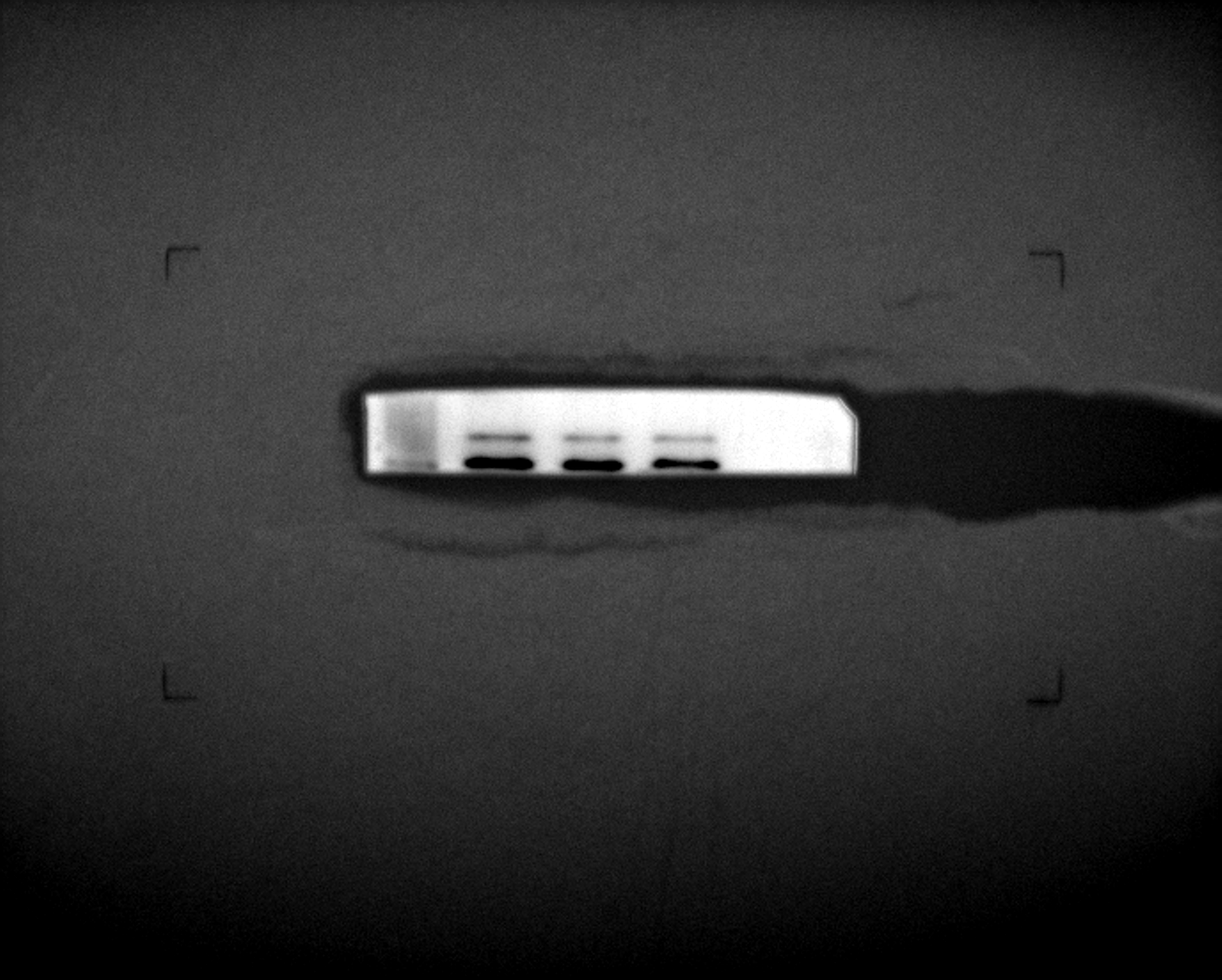

Supplement: Supplementary file 1 [file cancers-15-02356-s001.zip › file S1-Original Images for Blots/WB/ERK/ERK-vitro-2.tif]

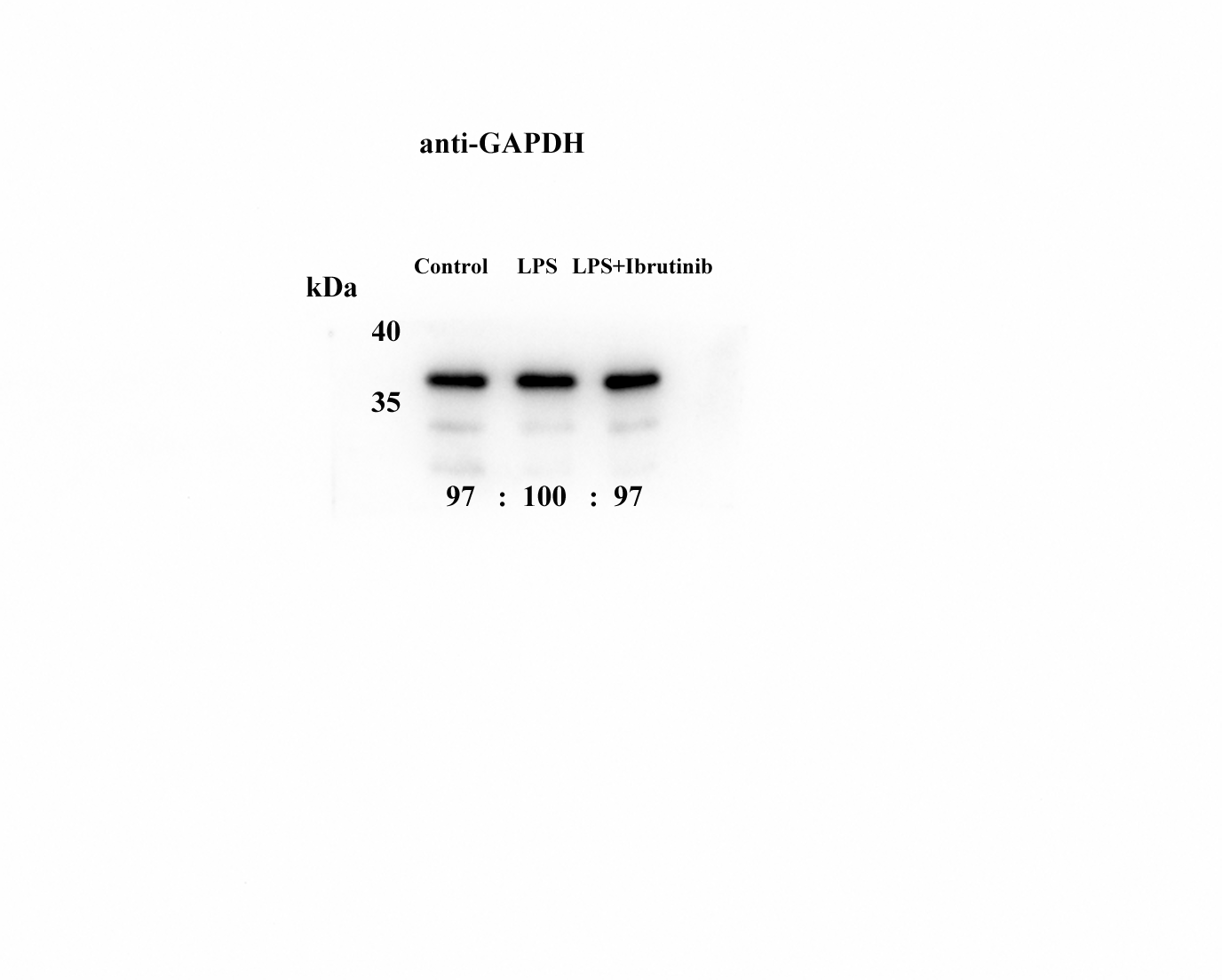

Supplement: Supplementary file 1 [file cancers-15-02356-s001.zip › file S1-Original Images for Blots/WB/GAPDH/GAPDH-I.tif]

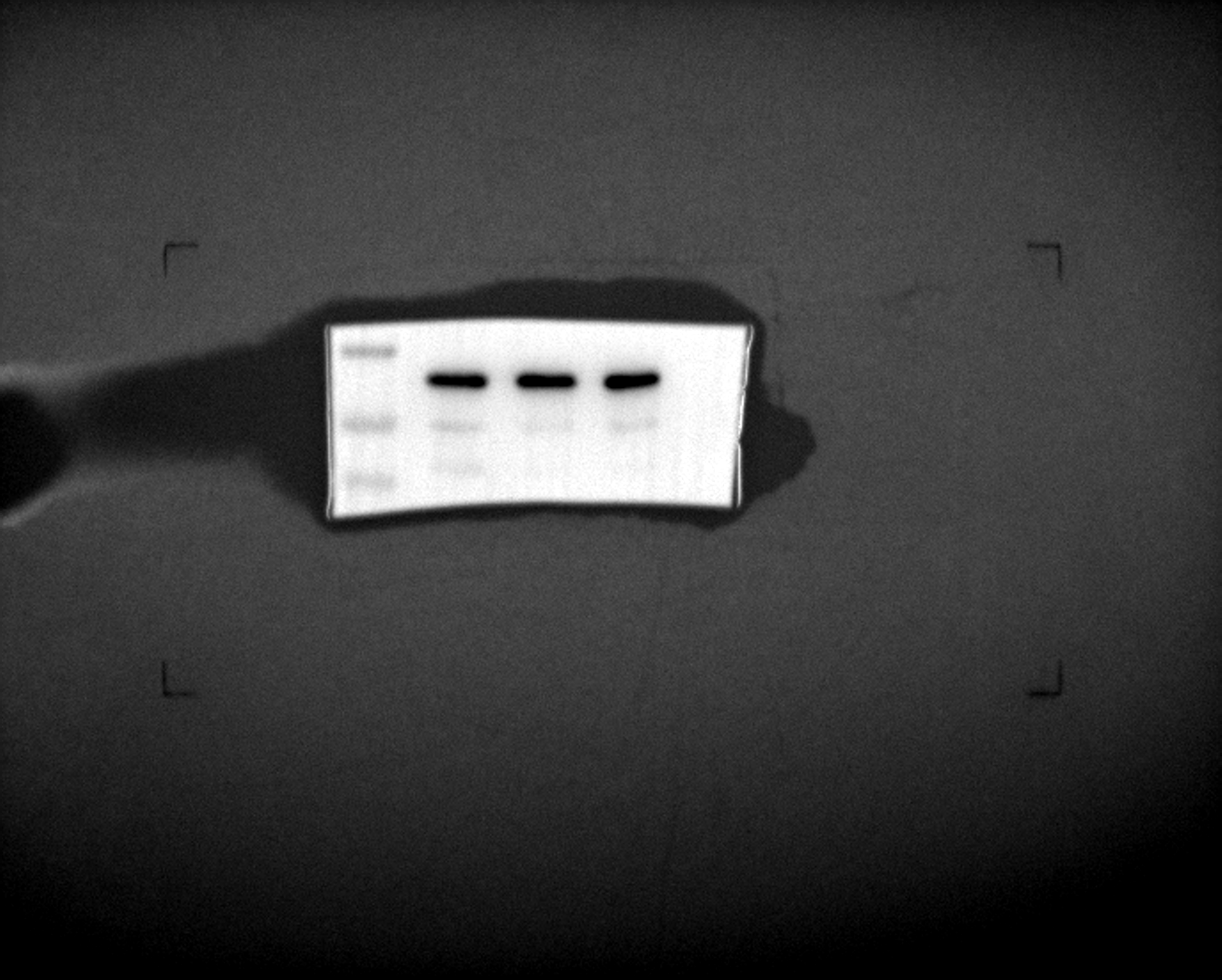

Supplement: Supplementary file 1 [file cancers-15-02356-s001.zip › file S1-Original Images for Blots/WB/GAPDH/GAPDH-J.tif]

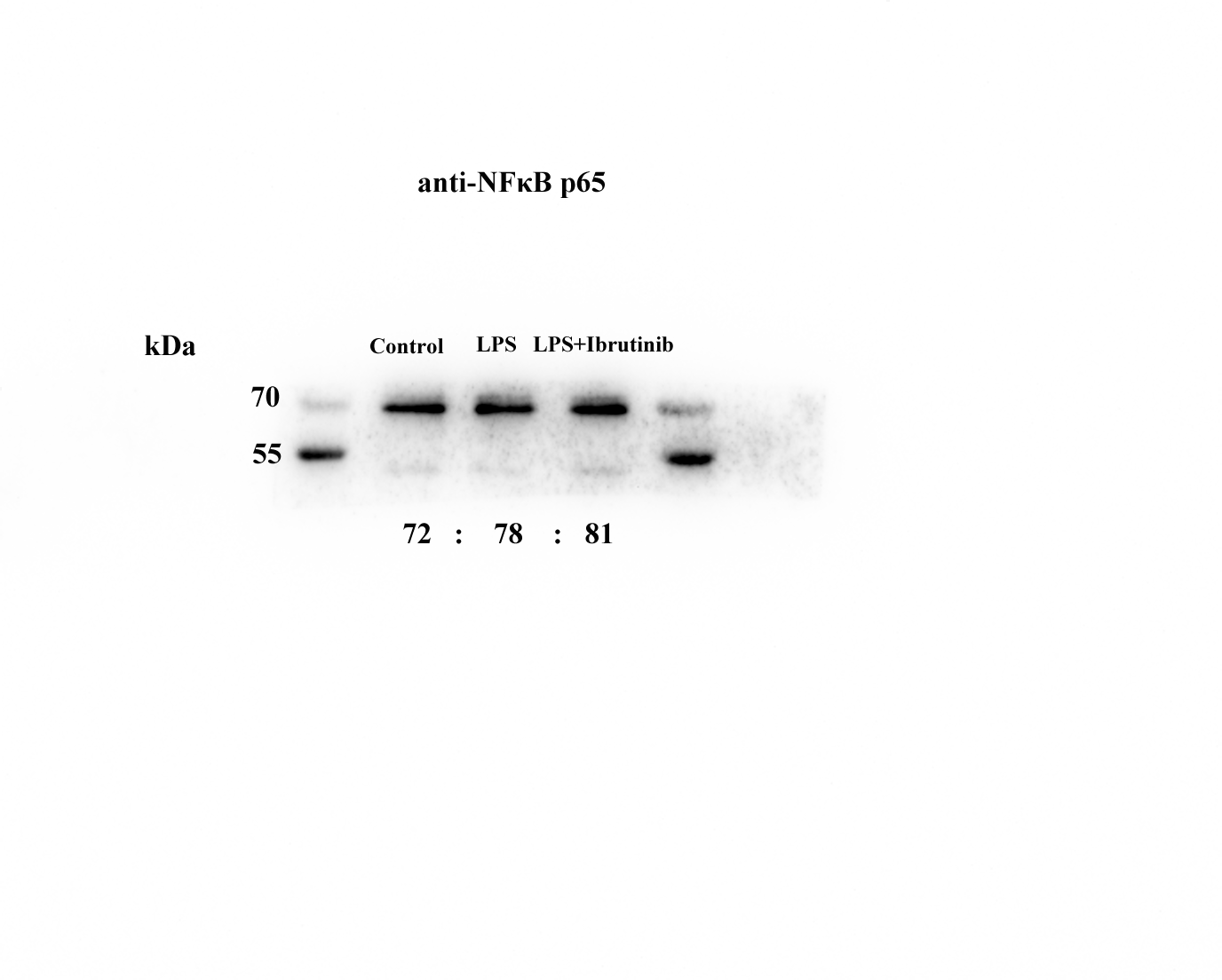

Supplement: Supplementary file 1 [file cancers-15-02356-s001.zip › file S1-Original Images for Blots/WB/NFKB/NFKB-C.tif]

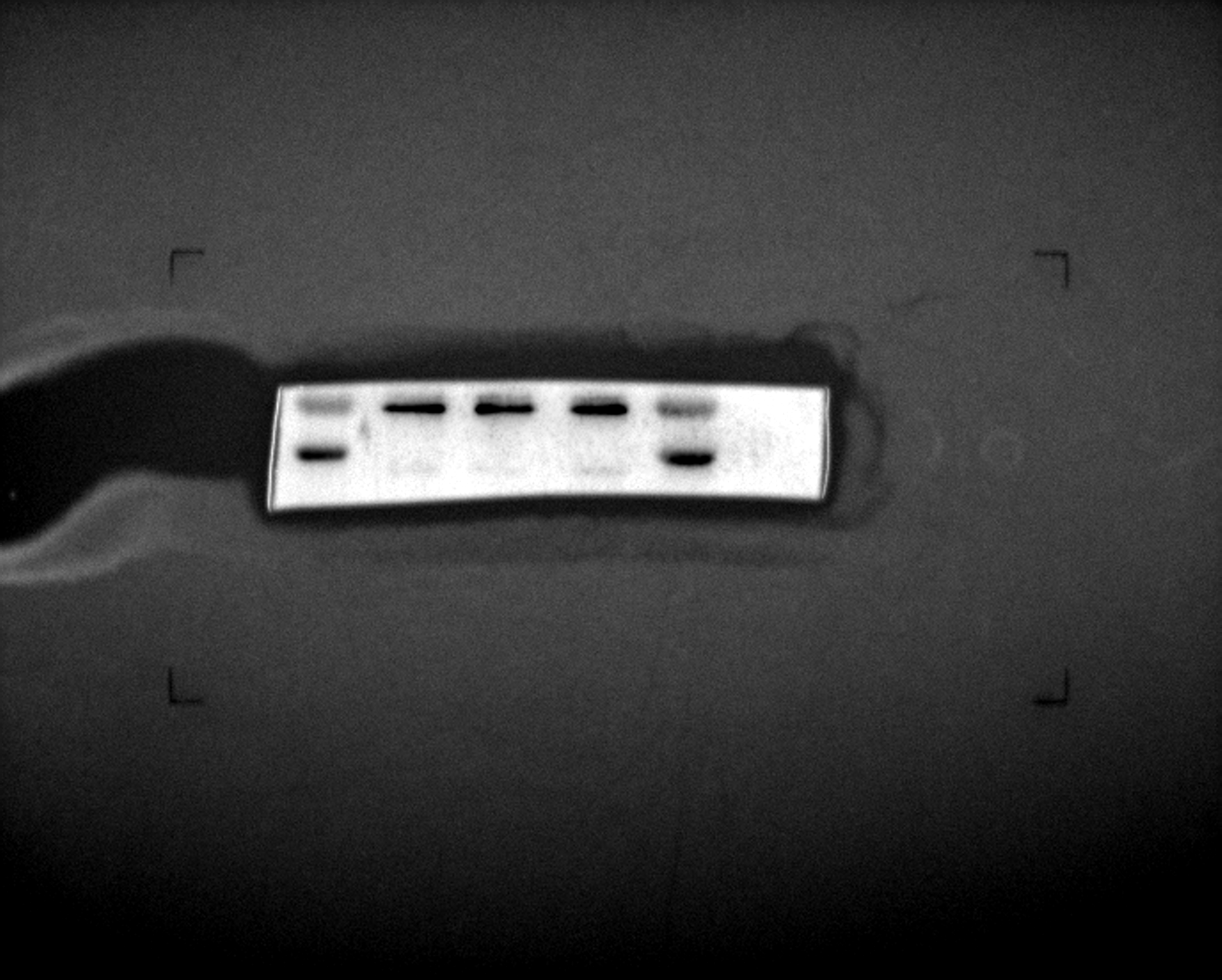

Supplement: Supplementary file 1 [file cancers-15-02356-s001.zip › file S1-Original Images for Blots/WB/NFKB/NFKB-D.tif]

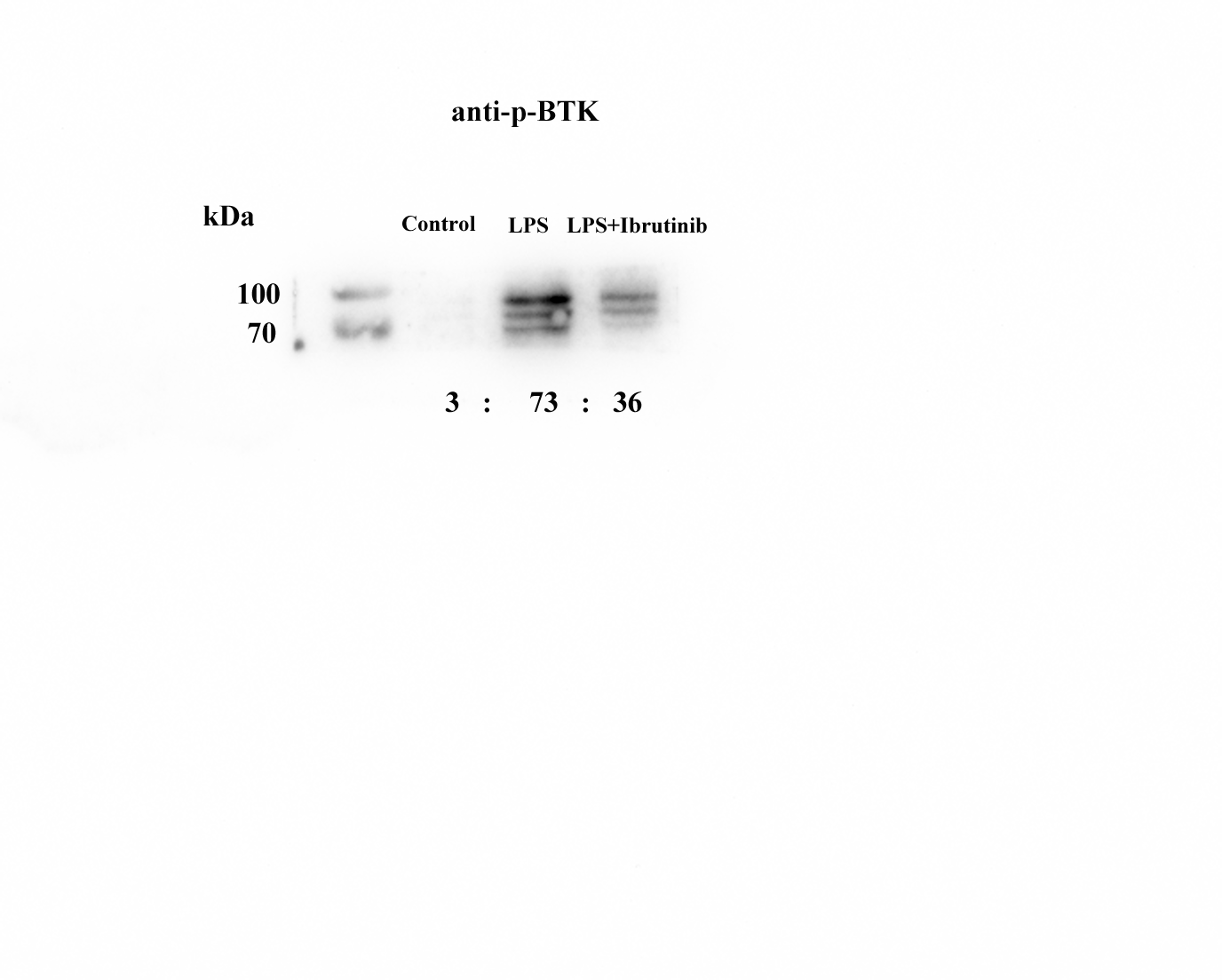

Supplement: Supplementary file 1 [file cancers-15-02356-s001.zip › file S1-Original Images for Blots/WB/p-BTK/p-BTK-E.tif]

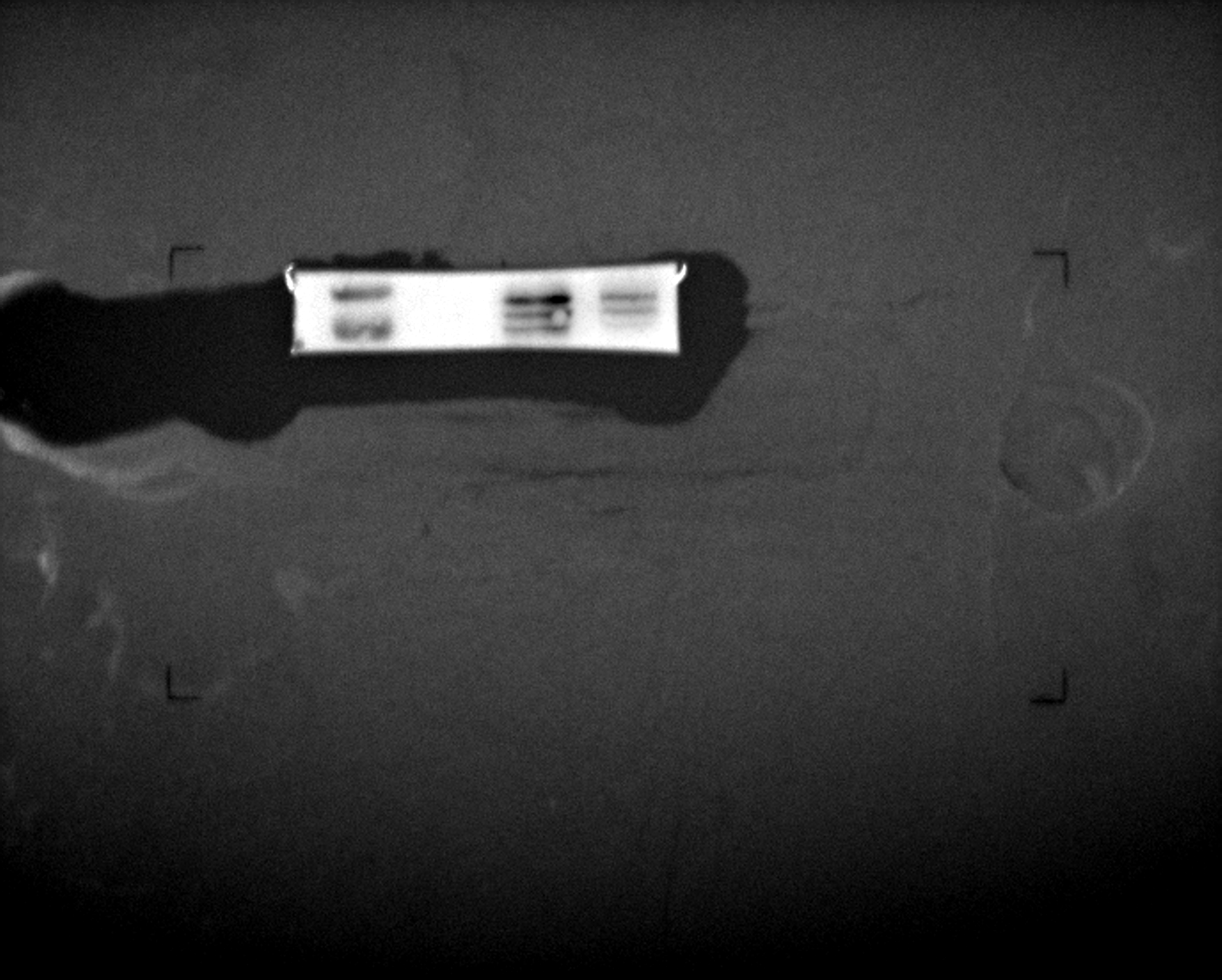

Supplement: Supplementary file 1 [file cancers-15-02356-s001.zip › file S1-Original Images for Blots/WB/p-BTK/p-BTK-F.tif]

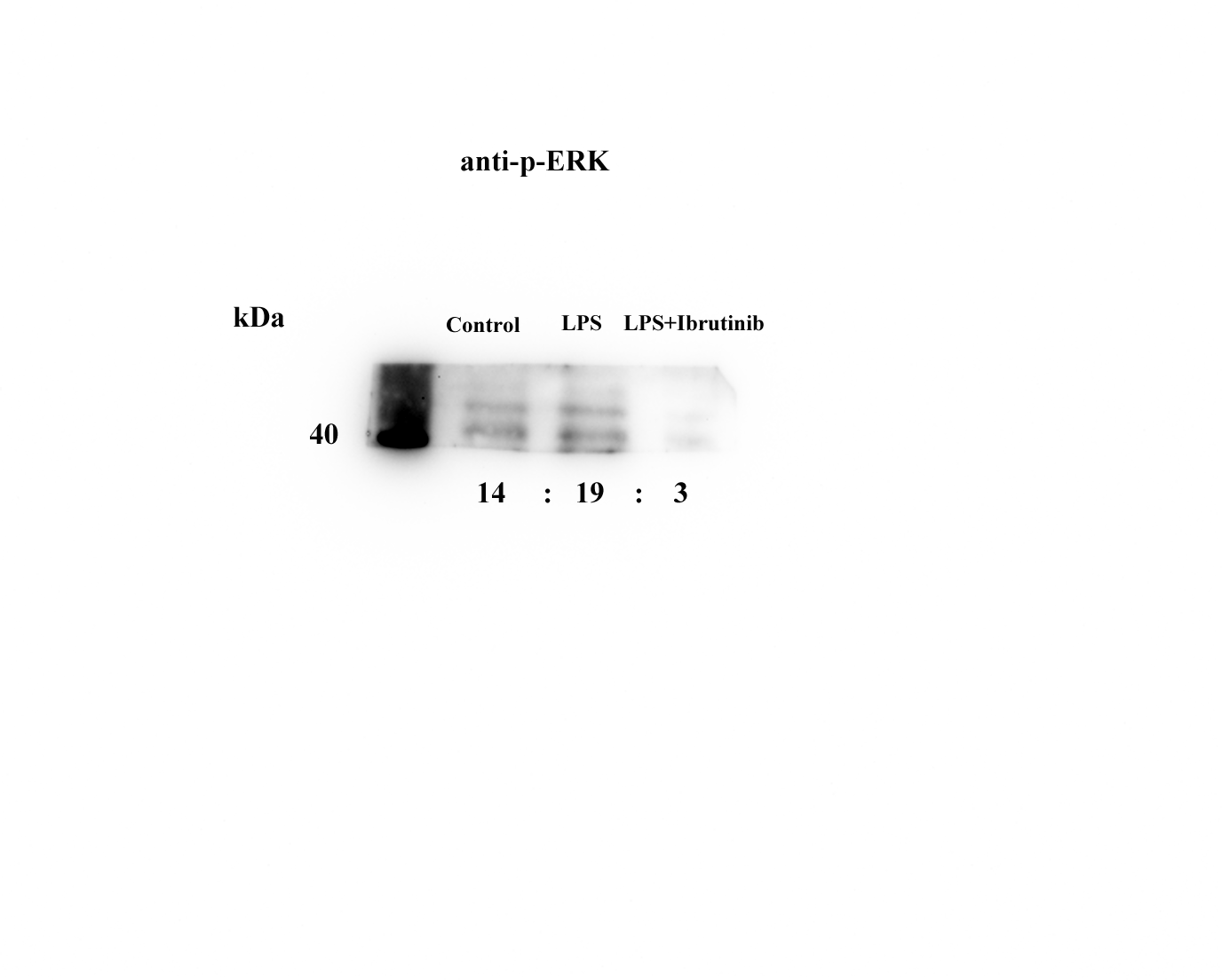

Supplement: Supplementary file 1 [file cancers-15-02356-s001.zip › file S1-Original Images for Blots/WB/p-ERK/pERK-vitro-1.tif]

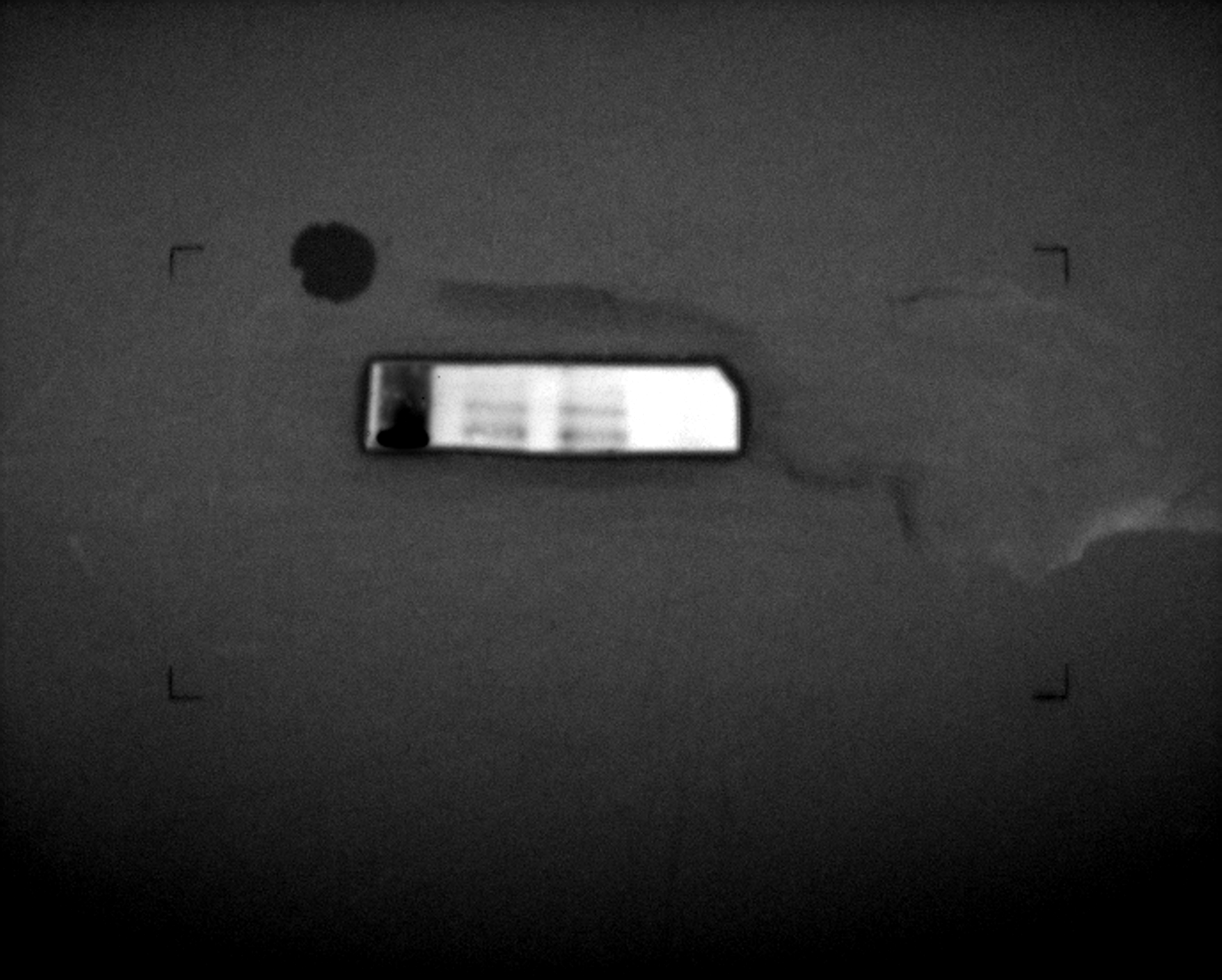

Supplement: Supplementary file 1 [file cancers-15-02356-s001.zip › file S1-Original Images for Blots/WB/p-ERK/pERK-vitro-2.tif]

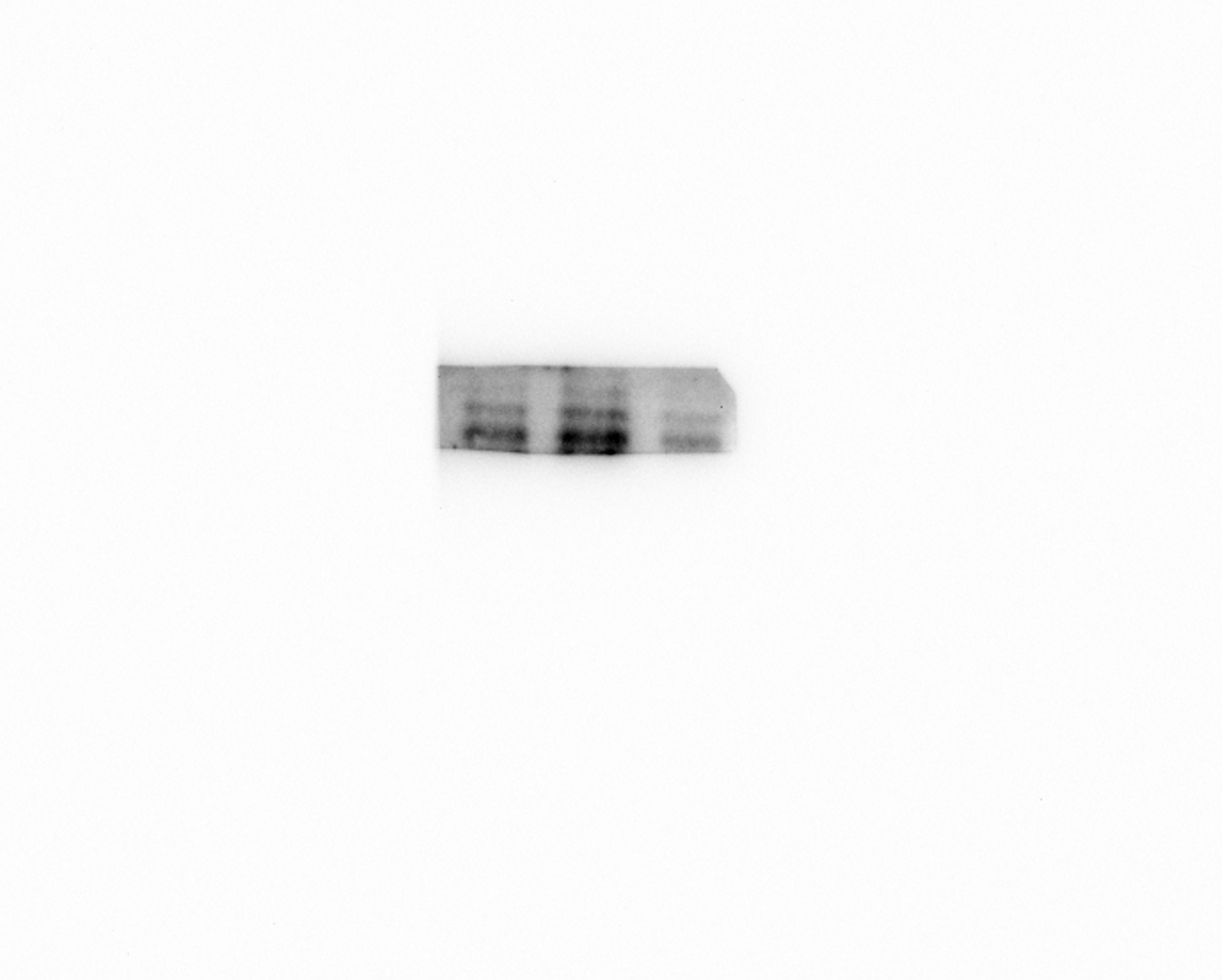

Supplement: Supplementary file 1 [file cancers-15-02356-s001.zip › file S1-Original Images for Blots/WB/p-ERK/pERK-vitro-3.tif]

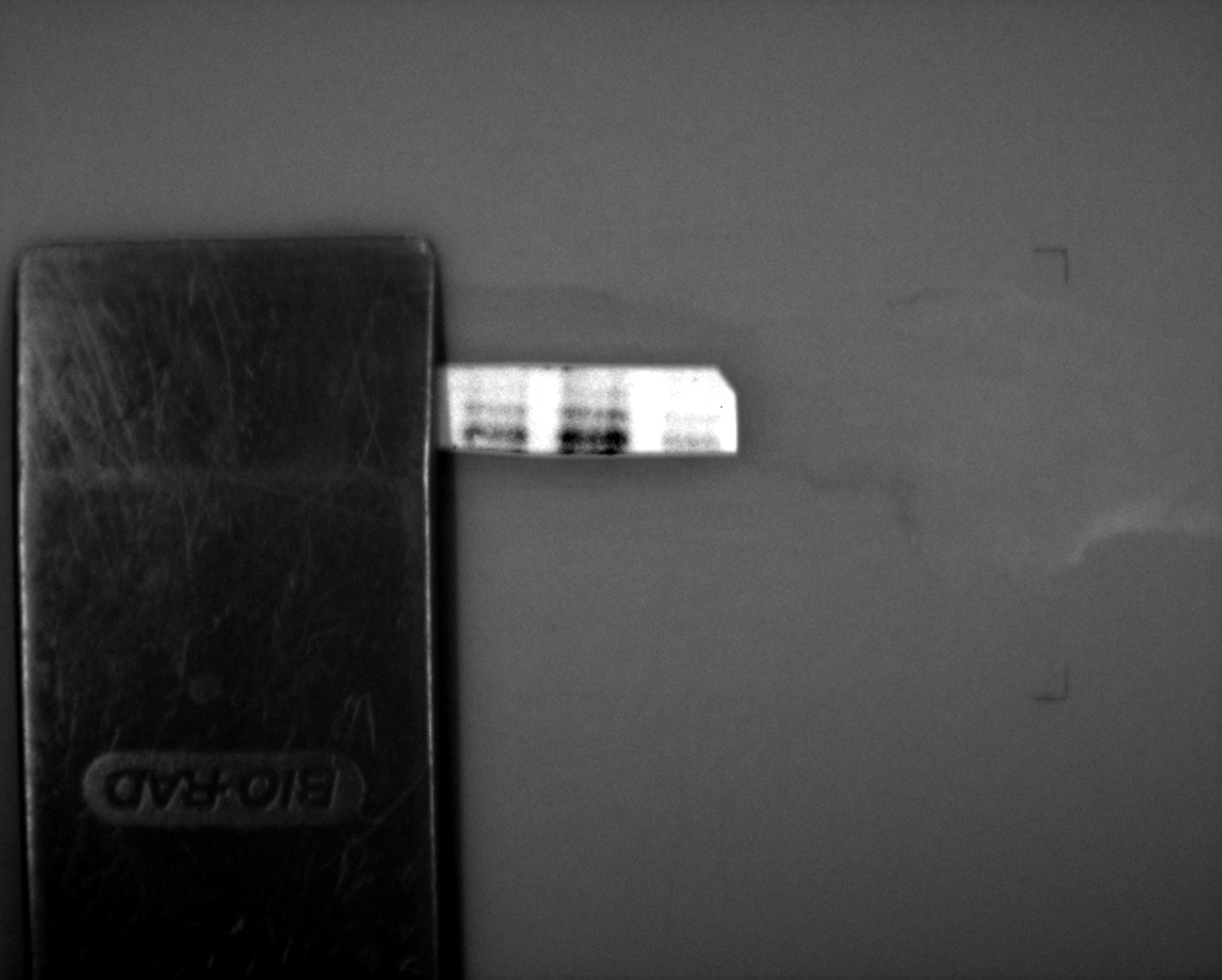

Supplement: Supplementary file 1 [file cancers-15-02356-s001.zip › file S1-Original Images for Blots/WB/p-ERK/pERK-vitro-4.tif]

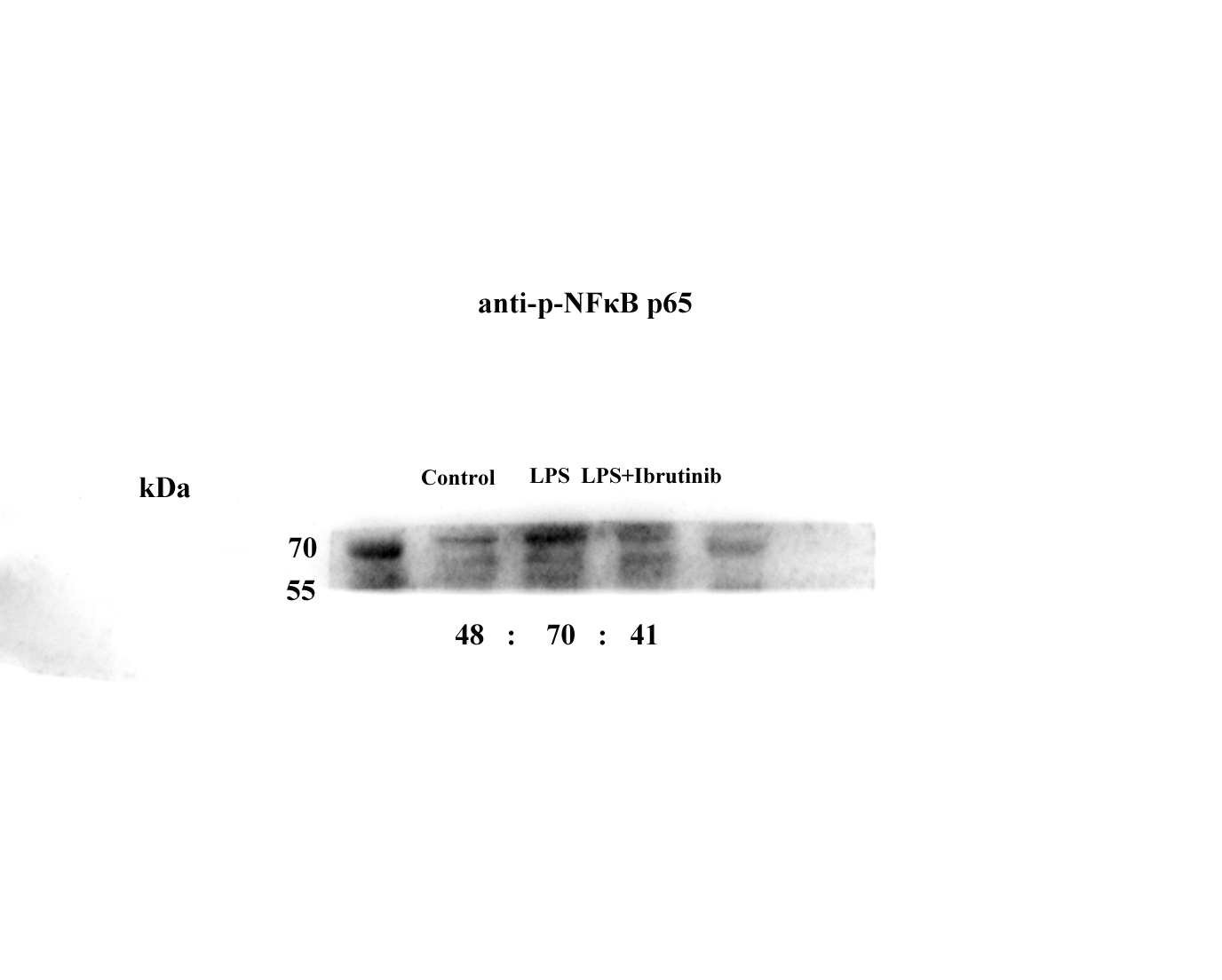

Supplement: Supplementary file 1 [file cancers-15-02356-s001.zip › file S1-Original Images for Blots/WB/p-NFKB/pNFKB-C.tif]

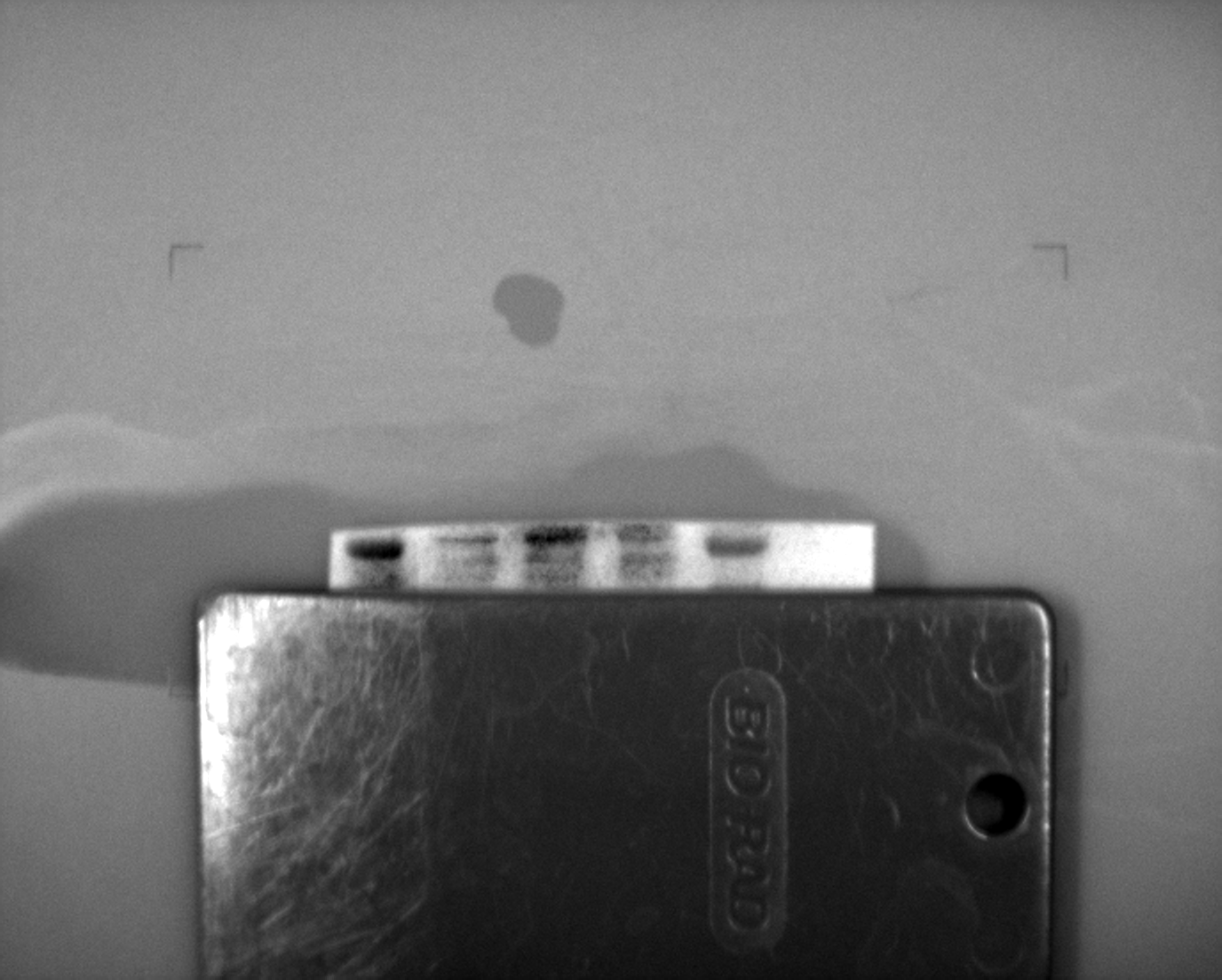

Supplement: Supplementary file 1 [file cancers-15-02356-s001.zip › file S1-Original Images for Blots/WB/p-NFKB/pNFKB-D.tif]

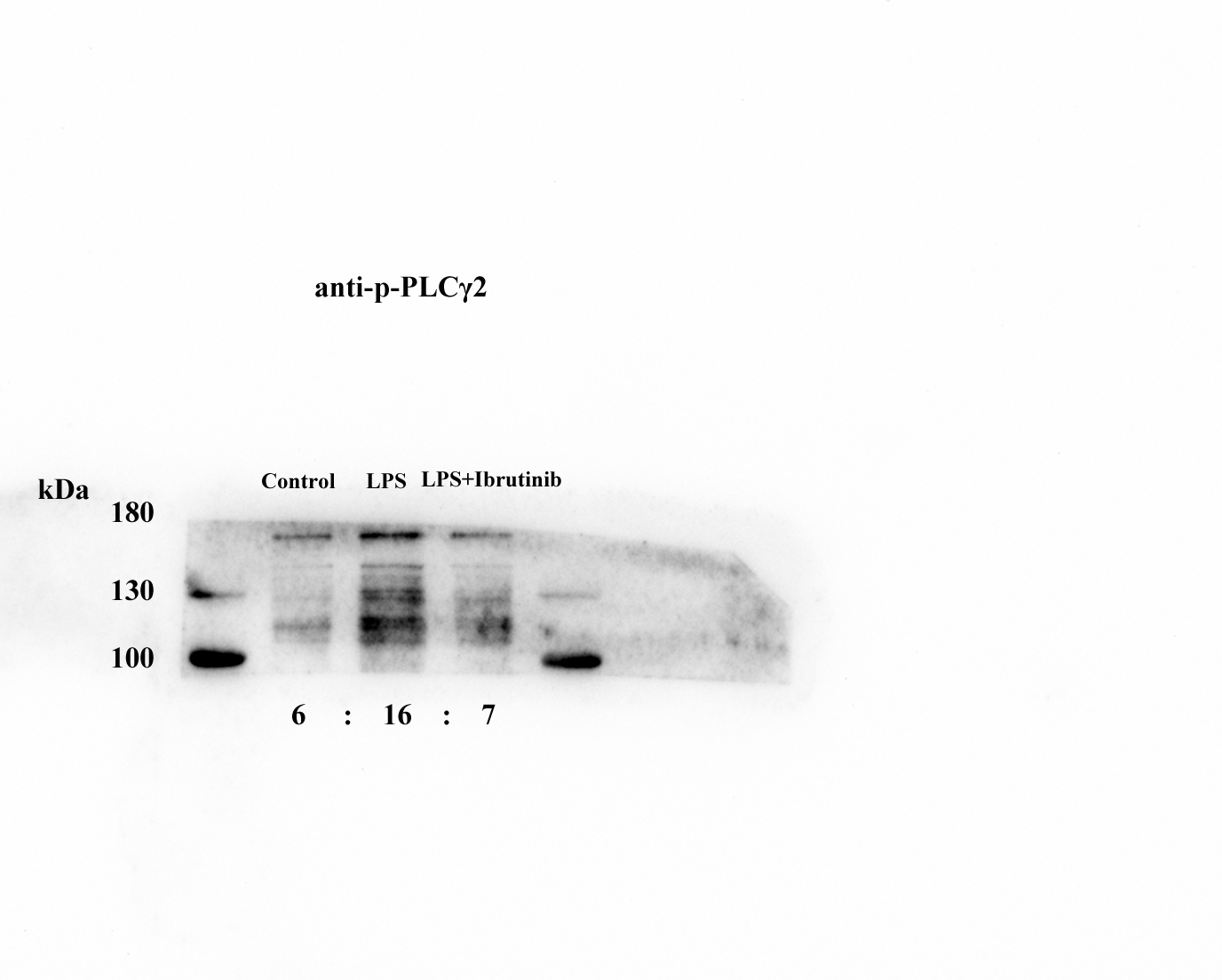

Supplement: Supplementary file 1 [file cancers-15-02356-s001.zip › file S1-Original Images for Blots/WB/p-PLGC2/pPLGC2-G.tif]

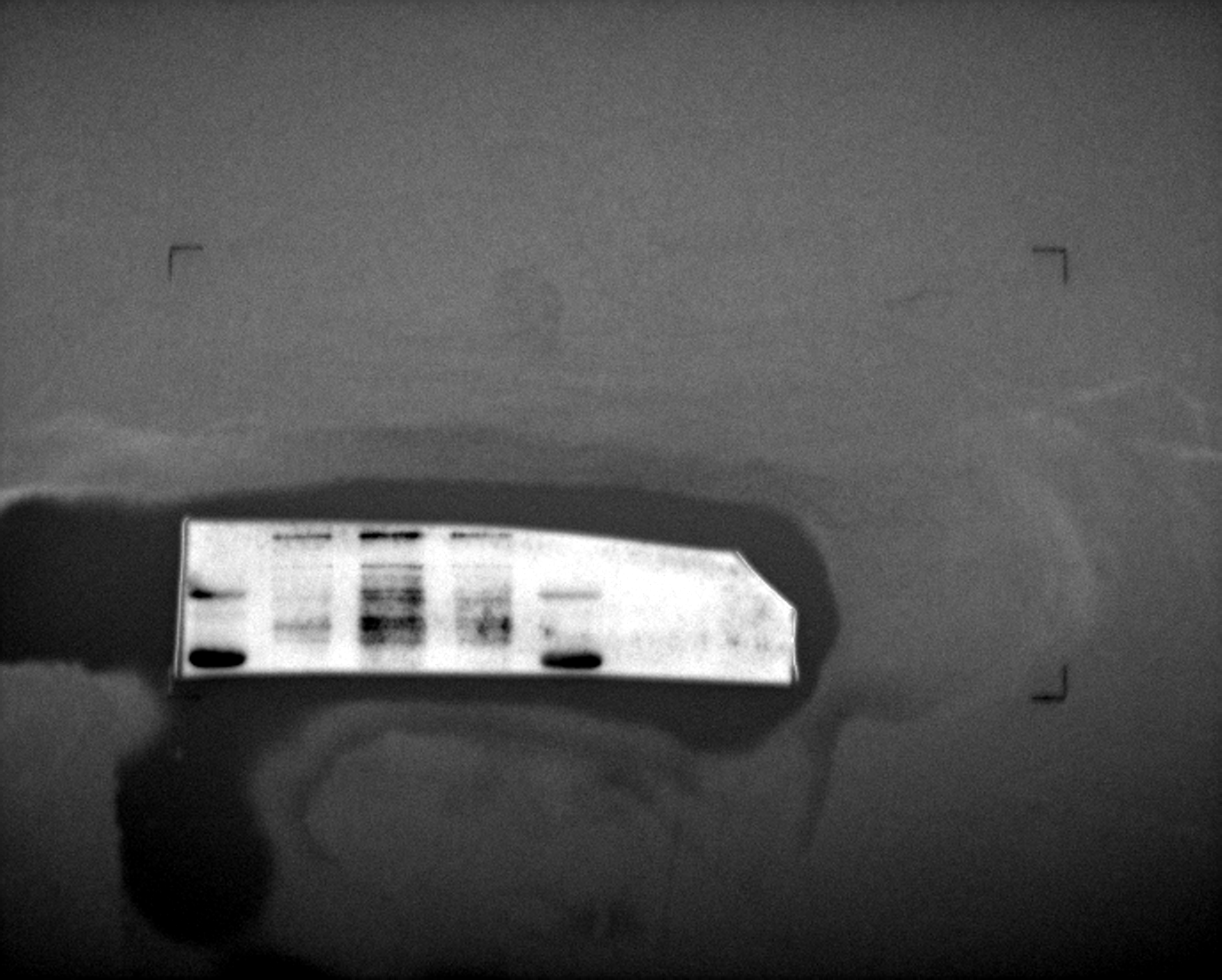

Supplement: Supplementary file 1 [file cancers-15-02356-s001.zip › file S1-Original Images for Blots/WB/p-PLGC2/pPLGC2-H.tif]

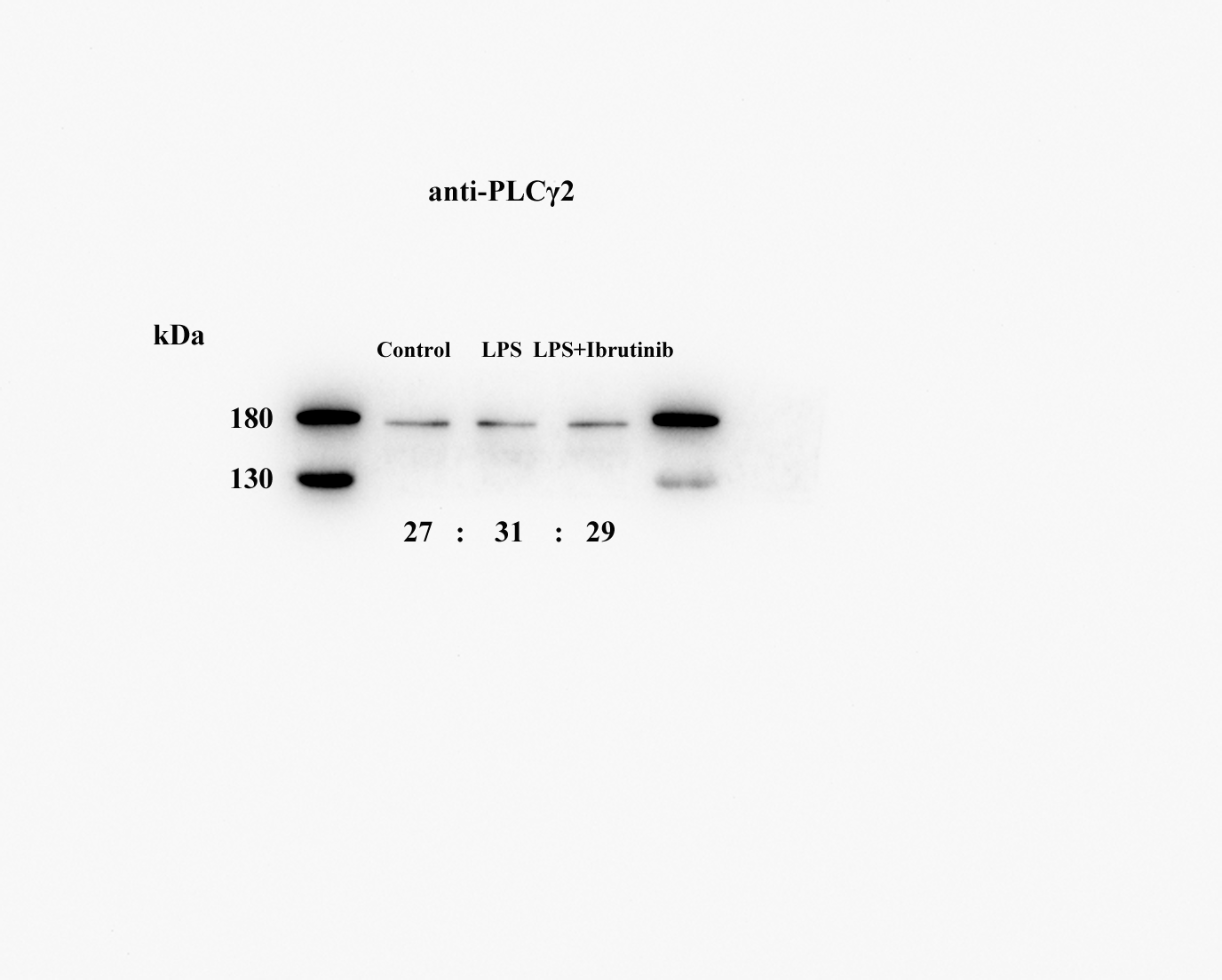

Supplement: Supplementary file 1 [file cancers-15-02356-s001.zip › file S1-Original Images for Blots/WB/PLGC2/PLGC2-C.tif]

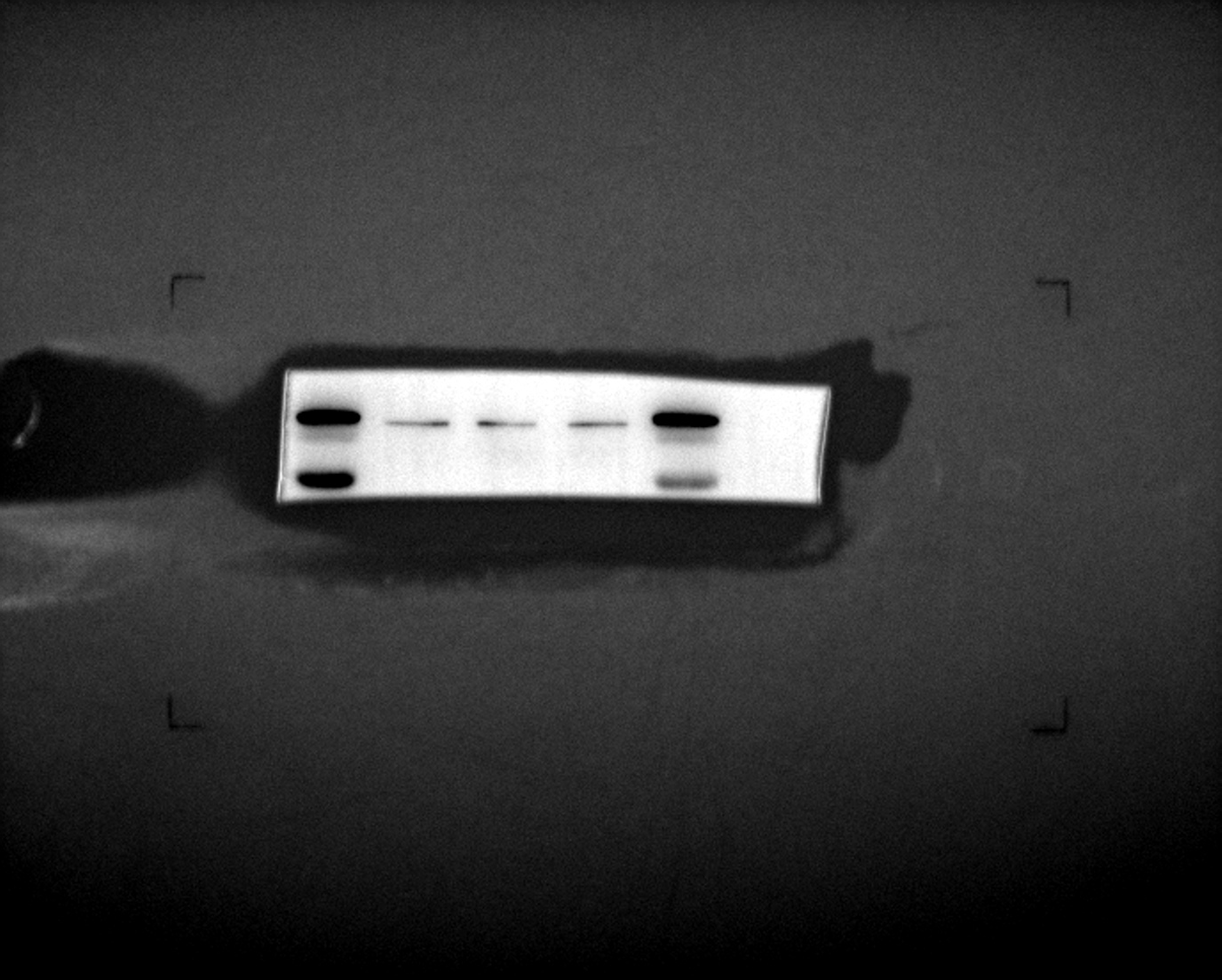

Supplement: Supplementary file 1 [file cancers-15-02356-s001.zip › file S1-Original Images for Blots/WB/PLGC2/PLGC2-D.tif]

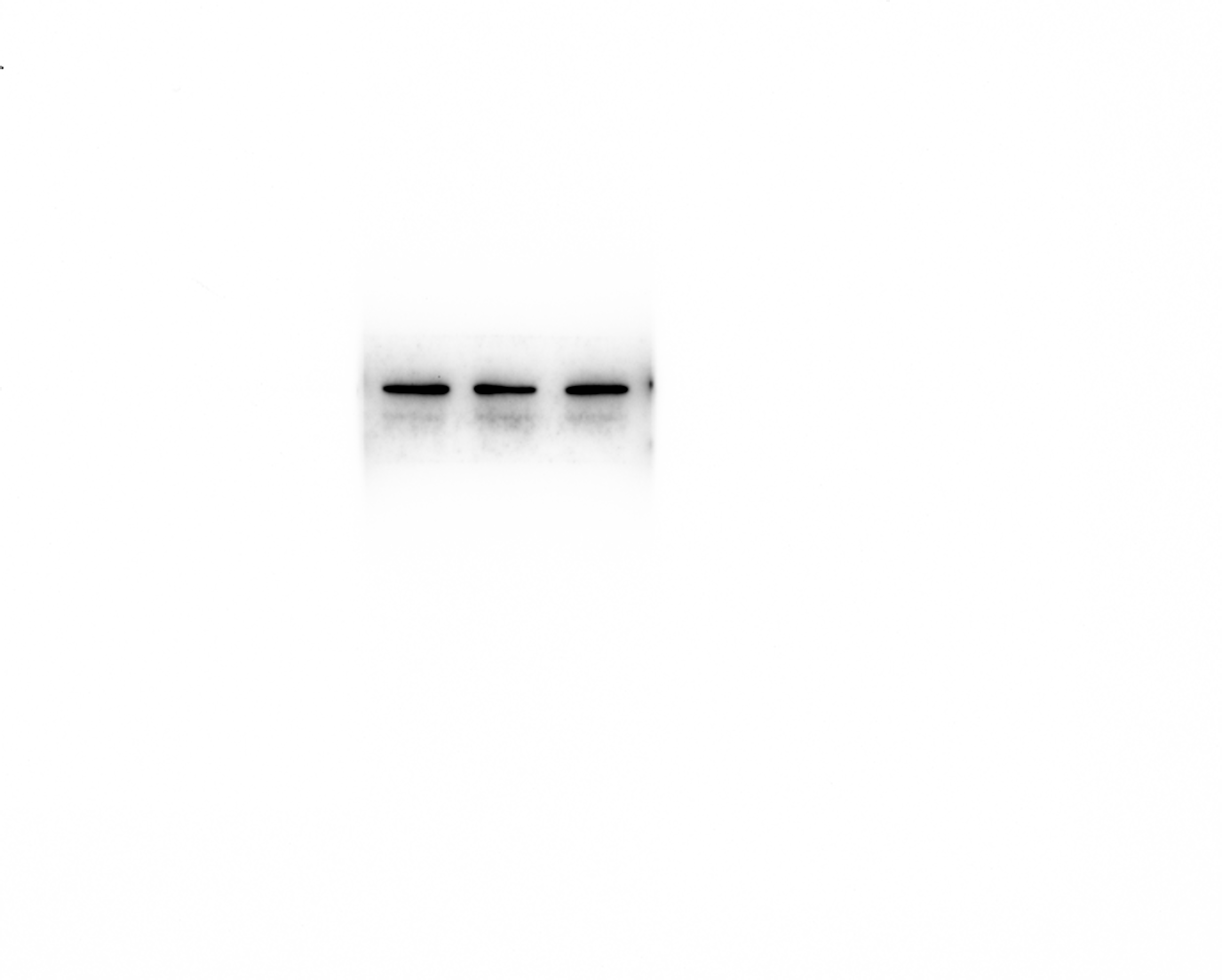

Supplement: Supplementary file 1 [file cancers-15-02356-s001.zip › file S1-Original Images for Blots/WB/PLGC2/PLGC2-E.tif]

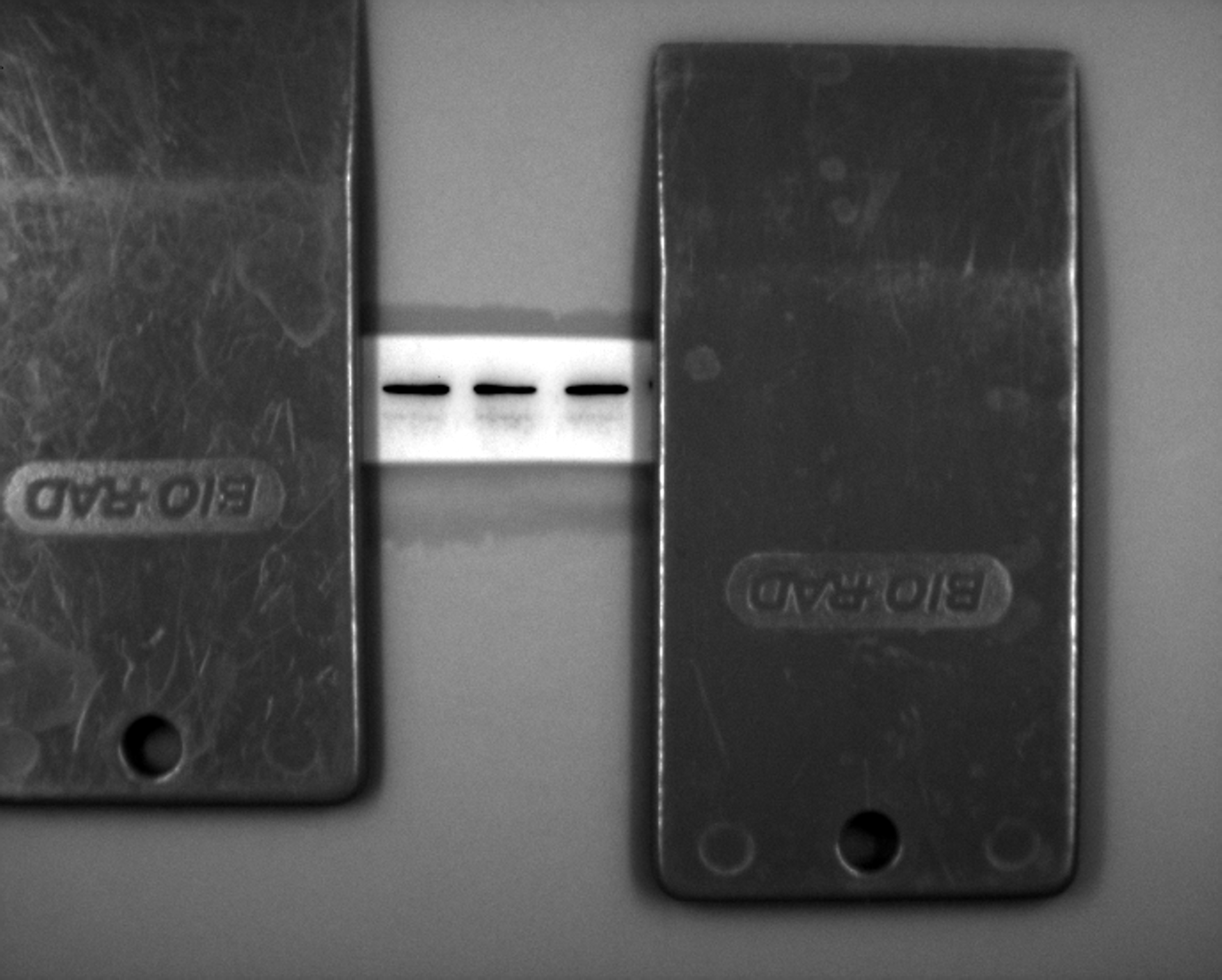

Supplement: Supplementary file 1 [file cancers-15-02356-s001.zip › file S1-Original Images for Blots/WB/PLGC2/PLGC2-F.tif]
